# The Emergence of Novel Benzodiazepines in Australia, Evidence, Alerts, Clinical Management and Harm Reduction—A Narrative Review

**DOI:** 10.1111/dar.70086

**Published:** 2025-12-07

**Authors:** Jack Freestone, Stassi Kypri, Jennifer L. Schumann, Erica Franklin, Cameron Francis, Monica J. Barratt, Amy Peacock, Rachel Sutherland, Brendan Clifford, Harriet MacDonald, Nadine Ezard, Kathryn Fletcher, Liam Acheson, Krista J. Siefried

**Affiliations:** ^1^ National Centre for Clinical Research on Emerging Drugs Sydney Australia; ^2^ National Drug and Alcohol Research Centre, UNSW Sydney Sydney Australia; ^3^ New South Wales Drug and Alcohol Clinical Research and Improvement Network, c/o the New South Wales Ministry of Health Sydney Australia; ^4^ Victorian Institute of Forensic Medicine Melbourne Australia; ^5^ Department of Forensic Medicine Monash University Melbourne Australia; ^6^ Monash Addiction Research Centre Melbourne Australia; ^7^ Pill Testing Australia Australia; ^8^ The Loop Australia Australia; ^9^ Criminology and Justice Studies, Social Equity Research Centre and Digital Ethnography Research Centre RMIT University Melbourne Australia; ^10^ Alcohol and Drug Service, St Vincent's Hospital Sydney Sydney Australia

## Abstract

**Issues:**

In Australia, detections of novel benzodiazepines (NBZ) and related overdoses have increased markedly over the last five years. This review summarises Australian peer‐reviewed literature and NBZ‐related drug alerts, outlines the pharmacology of commonly detected NBZs and discusses approaches to harm reduction, managing toxicity, dependence and withdrawal.

**Approach:**

Australian peer‐reviewed articles published between January 2020 and June 2025 were identified via Embase, PubMed, Scopus and PsycINFO. Drug alerts from this period were retrieved from an Australian online repository. Data were extracted, coded and synthesised. Global literature on the pharmacology associated with commonly detected NBZs in Australia, was retrieved via Google Scholar as were sources on benzodiazepine‐related harm reduction and clinical management and narratively summarised.

**Key Findings:**

Between 2020 and 2025, NBZs were frequently detected from data from emergency department, forensic, drug checking and coronial sources. The most common were etizolam, clonazolam, clobromazolam, bromazolam, flualprazolam and flubromazolam. Twenty‐three NBZ‐related alerts were issued over this period, with nearly half of these (*n* = 11) issued between January and June of 2025. Health responses are hindered by limited pharmacological data, detection challenges and little research on the experiences of consumers.

**Implications:**

To inform interventions spanning harm reduction and clinical management, future research must develop understandings of the pharmacology of NBZs and the experiences of people who source NBZs from unregulated markets.

**Conclusion:**

NBZs were consistently detected across Australian coronial, toxicological, forensic and drug checking data sources from 2020 to 2025. Their emergence represents a public health concern, worthy of ongoing attention and response.

## Key Point Summary

- Detections of novel benzodiazepines (NBZ) defined as unregulated, illicitly manufactured products containing compounds generally not approved for medical use, have increased in Australia and globally in recent years.
- This review summarises recent Australian peer‐reviewed literature and public drug alerts related to NBZs, while outlining pharmacological profiles of the most detected NBZs and discussing approaches to managing acute toxicity, dependence, withdrawal and harm reduction.
- NBZs are the most detected new psychoactive substance class in Australian emergency department data and were also frequently detected in forensic, coronial and drug checking data.
- Data indicate that etizolam, clonazolam, clobromazolam, bromazolam, flualprazolam and flubromazolam were the most frequently detected NBZs in Australia between 2020 and 2025.
- Effective health interventions for NBZs, including harm reduction education and support for dependence and withdrawal, are hindered by several challenges: limited pharmacological data on rapidly evolving compounds, inconsistent batch composition, difficulties in detection, and a poor understanding of the behaviors and characteristics of people who use NBZs.
- Future research should prioritise investigations into the pharmacology of NBZs, risks associated with counterfeit products, and the experiences, knowledge and harm reduction practices of people who use them.

## 1 Introduction

### 1.1 Benzodiazepine Use in Australia

As potent central nervous system (CNS) depressants, benzodiazepines induce sedation and are used to medically manage insomnia, anxiety, muscle spasms, seizures and alcohol withdrawal [1, 2]. When benzodiazepines are taken concomitantly with other CNS depressants or at high doses this may lead to potentially fatal toxicity [3, 4]. Prolonged use increases tolerance and the risk for dependence, and when discontinued, severe and potentially fatal withdrawal symptoms can occur [5, 6]. Given the risks associated with long‐term use of benzodiazepines, guidelines suggest that they be prescribed at low doses, intermittently, and over short periods [7].

Following opioids, benzodiazepines are the second most common drug class associated with overdose in Australia. In 2023, they were involved in 694 (39%) of such deaths highlighting a significant public health concern [8]. Between June 2023 and July 2024 in Australia, 4.5 million benzodiazepine scripts were dispensed to 1.4 million people [9]. However, the rate of benzodiazepine prescribing has declined over the last 10 years [9]. This decline followed the up‐scheduling of alprazolam from a Schedule 4 (prescription medication) to a Schedule 8 (controlled drug) in 2014, with further regulations introduced in 2017 when 2‐mg alprazolam tablets were delisted from public subsidy, alprazolam pack sizes were reduced from 50 tablets to 10 and refills were no longer allowed [10]. These regulations were accompanied by state and territory‐led initiatives to implement safer benzodiazepine prescribing guidelines and the Real Time Prescription Monitoring Program [7, 10, 11, 12]. Although the up‐scheduling of alprazolam led to a reduction in its use among people who inject drugs [13], it is speculated that increasing benzodiazepine regulation may lead some people to seek out non‐prescribed alternatives [11].

Non‐prescribed benzodiazepine use in Australia can include taking medications prescribed to others, in patterns which deviate from prescriber guidance, or using products obtained from unregulated markets [11]. Products sourced from unregulated markets can include: (i) medically diverted, legitimate pharmaceuticals from Australia; (ii) medically diverted pharmaceuticals from overseas; or (iii) illicitly manufactured counterfeits. According to the 2022–2023 National Drug Strategy Household Survey, 1.6% of Australians aged 14+ reported non‐medical use of tranquilisers or sleeping pills within the last 12 months [14]; this is likely to be a conservative estimate due to known underreporting of drug use in household surveys [15]. Among people who use drugs, the prevalence of non‐prescribed benzodiazepine use is substantially higher. Sentinel cross‐sectional studies show that 25% of people who regularly inject drugs and 28% of those who regularly use ecstasy or related drugs, reported using non‐prescribed benzodiazepines within the previous 6 months [16, 17]. Notably, in a study of people who reported non‐prescribed benzodiazepine use in the past 6 months, almost three‐fifths (59%) reported obtaining them via a diverted prescription [11].

### 1.2 What are NBZs?

Novel benzodiazepines (NBZ) refer to unregulated benzodiazepine analogues that are not approved for medical use in many jurisdictions and are often illicitly manufactured [5]. Worldwide, detections of NBZs have increased since 2019 [18, 19]. As of 31 December 2023, the European Union Drugs Agency was monitoring 36 distinct NBZ compounds [20], with this number likely to grow as new compounds emerge on the market. NBZs are commonly ingested orally and are often sold as counterfeit pharmaceuticals in tablet form, though they are also available as powders, liquids, gummies or blotters [21]. NBZs are similar in chemical composition to prescribed benzodiazepines, usually targeting the GABA_A_ receptor to induce anxiolytic, sedative, muscle relaxant, anticonvulsant and hypnotic effects [5]. However, they often demonstrate greater potency and/or have longer durations of effect than their pharmaceutical counterparts [22].

Counterfeit NBZs are frequently designed to mimic brand‐name medicines but may contain unknown substances or inconsistent dosages, making them difficult to identify and their pharmacological effects unpredictable. Dosage and strength can also vary significantly between products or even within a single batch [23]. In some cases, a single tablet may contain multiple NBZ compounds [24]. This unpredictability poses substantial risks, particularly if consumers believe they are taking known quantities of regulated benzodiazepines. Adverse effects can include intense sedation, amnesia and respiratory depression [24, 25, 26]. Compared to pharmaceutical benzodiazepines, NBZs are associated with greater risks of acute toxicity and severe withdrawal [5]. Data from the Emerging Drugs Network of Australia indicate that NBZs are now the most commonly identified new psychoactive substances (NPS) in toxicology monitoring programs carried out in emergency departments [27].

### 1.3 Aims

Despite growing concerns, there has been no synthesis of the evidence around the emergence of NBZs in Australia. This narrative review was systematically conducted to: (i) summarise recent peer‐reviewed literature and public drug alerts regarding detected NBZs in Australia; (ii) provide a high‐level overview of existing information on the pharmacodynamic and pharmacokinetic profiles of some of the commonly detected NBZs in Australia; (iii) describe the management of acute NBZ toxicity, withdrawal and dependence; and (iv) outline priorities for NBZ‐related harm reduction education.

## 2 Methods

Given the breadth of aims outlined above, a narrative review methodology was selected. As outlined below, search, extraction and synthesis activities were first conducted to identify relevant recent Australian sources (aim 1) with a supplementary search and synthesis process conducted to address aims 2, 3 and 4.

### 2.1 Search and Screening

In alignment with the methodological guidance for the systematic and transparent conduct of narrative reviews [28, 29] a search for literature to address research aim 1 was conducted by adopting search protocols and eligibility criteria commonly used in systematic reviews. A search for Australian peer‐reviewed literature and public drug alerts on NBZs, was led by the first author JF with support from SK and HM. Peer‐reviewed literature was identified through systematic searches of EMBASE, PubMed, Scopus, and PsycINFO. Search terms related to three overarching concepts: (i) novel/new benzodiazepines; (ii) drug monitoring systems and associated data sources; and (iii) Australia. A full list of search terms is provided as Supporting Information. Public drug alerts were sourced via a retrospective review of a database used to populate theknow.org.au, an online repository of public drug warnings issued across every Australian state and territory [30, 31].

Following the search for Australian publications, supplementary global evidence related to NBZ pharmacology, clinical management and harm reduction education (research aims 2, 3 and 4) was obtained through a Google Scholar search of peer‐reviewed and grey literature. Specific searches for each of the NBZs commonly identified in Australian peer‐reviewed literature and Australian drug alerts were conducted, as were searches pertaining to the management of benzodiazepine toxicity, dependence and withdrawal.

Peer reviewed literature and drug alerts were screened for inclusion by JF, with decisions around inclusion and exclusion supported by SK and KJS. The list of included Australian peer‐reviewed studies and drug alerts was later reviewed by all authors.

### 2.2 Eligibility

Inclusion of Australian evidence addressing research aim 1 was limited to original studies where NBZ‐related findings were a primary focus and based on Australian data collected between January 2020 and June 2025. Drug alerts were included if they were issued during the same period and addressed acute health risks linked to NBZs. The 2020 to 2025 period was selected in alignment with the aim to generate an understanding of contemporary Australian evidence and to concord with the period in which capacity for monitoring of unregulated drug markets and associated harms in Australia was bolstered by the establishment of additional mechanisms to monitor drug markets, identify concerns and issue drug alerts [32, 33, 34, 35, 36, 37].

Supplementary literature retrieved to inform aims 2, 3 and 4 were not subject to the same eligibility criteria. However, these sources were included if they presented empirical evidence regarding the pharmacology of commonly detected NBZs, approaches to managing toxicity, dependence, withdrawal or harm reduction.

### 2.3 Data Extraction and Synthesis

To address research aim 1, data extraction was led by author JF, who coded studies and drug alerts in alignment with a coding framework. This framework was reviewed and coding checked by authors NE, SK, HM, LA, BC, KF, and KJS. Data from supplementary global evidence outlining the pharmacology of commonly detected NBZs (research aim 2) was extracted and double coded by JF and SK in accordance with the coding framework addressing pharmacokinetics and key effects. Australian and internationally peer‐reviewed and grey literature was then narratively synthesised to specifically address research aim 3 regarding clinical management of toxicity, dependence or withdrawal, and research aim 4, harm reduction strategies.

## 3 Results

### 3.1 Research Aim 1: Summarising Recent Peer‐Reviewed Literature and Public Drug Alerts on NBZs in Australia

Table 1 outlines findings from 14 peer‐reviewed publications presenting original Australian data relating to analytically detected, administratively confirmed or self‐reported use of NBZs. Of the studies included in Table 1, seven presented data from Australian emergency departments [3, 4, 24, 38, 39, 40, 41], four of which were cross‐sectional studies that measured the frequency of NBZ detection [3, 4, 38, 39] and three were case series outlining the clinical characteristics of patients presenting with acute NBZ toxicity [24, 40, 41]. Additionally, three studies were coronial reviews of NBZ‐related mortality [19, 42, 43], two predominantly reported on forensic analysis of seized samples [23, 46], one was a wastewater analysis [45] and one was a cross‐sectional survey of people reporting recent non‐prescribed benzodiazepine use [11].

**TABLE 1:** Australian peer reviewed publications focussed on NBZs between January 2020 and June 2025.

First author, year Jurisdiction [ref]	Title	Study type data source data collection dates	Sample parameters	NBZs detected^a^	Key findings relating to NBZs
Emergency department toxicology, cross sectional studies (*n* = 4)					
Castle, 2024 Victoria [38]	Identification of clobromazolam in Australian emergency department intoxications using data‐independent high‐resolution mass spectrometry and the HighResNPS.com database	EDNA‐V April 2022 to 31 March 2023	*N* = 993 clinical cases analysed part of the Emerging Drugs Network of Australia—Victoria (EDNA‐V) project from 1 April 2022 to 31 March 2023	Bromazolam, Clobromazolam, Clonazolam Deschloroetizolam, Estazolam, Etizolam, Flualprazolam, Flubromazepam, Phenazepam	Clobromazolam was the most detected NBZ, identified in 100 out of 993 cases. No patient reported deliberately using clobromazolam, more than half indicated alprazolam use but it was only found in 7% of cases. Among the 100 clobramazolam cases, phenazepam was co‐detected in *n* = 45, methylamphetamine in *n* = 71 and other benzodiazepines in *n* = 60.
Weber, 2023 Western Australia [4]	Analytically confirmed illicit and novel psychoactive drug use in Western Australian emergency departments: initial results from the Emerging Drugs Network of Australia (EDNA)	EDNA WA Apr 2020 ‐ Dec 2021	*N* = 434 patients presenting with unusual clinical features consistent with drug toxicity across five Western Australian emergency departments	Bromazolam, Clonazolam, Etizolam, Flualprazolam, Flubromazolam	48 NPS were detected across 43 distinct presentations. Among these 43 presentations, NBZs were detected in *n* = 24. The most detected NBZ was clonazolam (*n* = 12) followed by flualprazolam and etizolam which were each detected across *n* = 7 distinct presentations.
Alfred, 2023 South Australia [39]	The South Australian Emergency Department Admission Blood Psychoactive Testing (EDABPT) program: first results	EDABPT program Mar 2019—May 2020	*N* = 1120 eligible patients aged 18 years and over, presenting to 4 metropolitan SA EDs	Etizolam Flualprazolam Flubromazolam	29.6% of presentations contained one or more benzodiazepine. Authors note concerns regarding number of samples with concurrent detections of diazepam, pregabalin and opiate agonists. Increases in benzodiazepine type NPS were noted towards the end of the study period (May 2020).
Syrjanen, 2023 Victoria [3]	Characteristics and time course of benzodiazepine type new psychoactive substance detections in Australia: results from the Emerging Drugs Network of Australia—Victoria project 2020–2022	EDNA‐V Sep 2020—Aug 2022	*N* = 1112 cases in the EDNA‐V centralised registry of which *n* = 183 had analytical confirmation of a benzodiazepine‐type NPS exposure	Bromazolam, Clobromazolam, Clonazolam, Desalkylflurazepam, Deschloroetizolam, Diclazepam, Estazolam, Etizolam, Flualprazolam, Flubromazepam, Flubromazolam, Phenazepam	Twelve different benzodiazepine‐type NPS were detected over a two‐year period, the two most common being clonazolam and etizolam. Patients were predominantly young males. No patients reported the use of benzodiazepine type NPS by name indicating that people who source benzodiazepines from unregulated markets may not be aware of the large variety in benzodiazepine type NPS.
Emergency department case series (*n* = 3)					
Partridge, 2024 South Australia [40]	A cluster of multi‐drug intoxications involving xylazine, benzimidazole opioids (nitazenes) and novel benzodiazepines in South Australia	EDNA‐SA Dec 2023	*N* = 3 patients, presenting to the Royal Adelaide Hospital	Bromazolam, Nitrazolam	Toxicological analysis of blood samples from *n* = 3 patients identified xylazine, protonitazene, metonitazene, bromazolam and nitrazolam. This paper documents the first instance of nitrazolam use in Australia.
Syrjanen, 2023 Victoria [24]	From signal to alert: A cluster of exposures to counterfeit alprazolam tablets containing five novel benzodiazepines	EDNA‐V *April 2022*	*N* = 6 patients (1 female, 5 males aged 17–45 years, presenting to EDs), *n* = 2 reporting use of counterfeit Mylan and *n* = 4 reporting use of counterfeit Xanax	Bromazolam, Clonazolam, Etizolam, Flualprazolam, Flubromazepam	Toxicological analyses identified five separate NBZs within blood samples of six patients presenting with prolonged sedation. Exposures were related to the sale of counterfeit alprazolam products branded ‘Mylan’ and ‘Xanax’.
Syrjanen, 2023 Victoria [41]	Non‐fatal intoxications involving the novel benzodiazepine clonazolam: case series from the Emerging Drugs Network of Australia – Victoria project	EDNA‐V *Dates not reported*	*N* = 4 presenting to Victorian emergency departments, *n* = 3 of whom self‐reported use of counterfeit Xanax/alprazolam	Clonazolam	Patients presented with a sedative toxidrome following exposure to Xanax (a brand not available in Australia). Median time to return to normal consciousness was 23 h. This case series led to a clinician alert about counterfeit benzodiazepines and the possibility of prolonged sedation after exposure.
Coronial studies (*n* = 3)					
Schumann, 2025 Victoria [19]	Changes over time in novel benzodiazepines contributing to fatal overdoses in Victoria, Australia	Victorian overdose deaths register January 2009—December 2023	*N* = 140 NBZ‐involved overdose deaths in Victoria	Bromazolam, Clobromazolam, Clonazolam, Delorazepam, Desalkylflurazepam, Desalkylglidazepam, Deschloroetizolam, Diclazepam, Estazolam, Etizolam, Flualprazolam, Flubromazepam, Flubromazolam, Lormetazepam, Phenazepam	Between 2015 and 2023, a total of 140 deaths were recorded in which an NBZ was detected. The first identified death occurred in 2015, but NBZ related fatalities remained rare until 2019, with only two cases recorded prior. Annual deaths then increased, peaking in 2022 with 40 cases. In every case, at least one additional substance contributed to death. The NBZs most frequently involved were etizolam (*n* = 60 deaths), bromazolam (*n* = 39 deaths), clonazolam (*n* = 34 deaths), flualprazolam (*n* = 33 deaths), and clobromazolam (*n* = 21 deaths).
Drummer, 2024 Victoria [42]	Deaths involving novel benzodiazepines in Victoria, Australia from 2018 to 2022	Victorian coronial cases January 2018 – December 2022	*N* = 133 cases in which NBZs were reported in Victorian coronial cases	Bromazolam, Clonazolam, Clobromazolam, Desalkylflurazepam, Diclazepam, Estazolam, Etizolam, Flualprazolam, Flubromazolam, Flubromazepam, Phenazepam	Detection patterns shifted over the five‐year period, with newer NBZs like bromazolam, clobromazolam, flubromazepam, and phenazepam emerging in the more recent data, while etizolam was consistently detected since 2018. Of *n* = 133 deaths, *n* = 95 were deemed drug‐related, all involving other CNS depressants. Many cases featured multiple NBZs, with five or more benzodiazepines detected in eight instances.
Darke, 2022 National [43]	Characteristics of fatal ‘novel’ benzodiazepine toxicity in Australia	NCIS 2000–2021	A total of 40 cases were identified (the first occurring in 2015)	Delorazepam, Diclazepam, Etizolam, Flubromazolam, Flubromazepam, Flualprazolam, Lormetazepam	Of the 40 NBZs deaths identified since 2015 the median age of those who died was 26.5 years and 87.5% were male. Death was due to accidental toxicity in 92.5% of cases. Etizolam was the most common NBZ detected (87.5%). A CNS depressant other than a NBZ was detected in 95.0% of cases.
Analysis of seized samples (*n* = 2)					
Smith, 2025 New South Wales [44]	Contents and time‐course of falsified alprazolam detections in New South Wales, Australia.	Retrospective review of police seizures in New South Wales + analysis of substances acquired from New South Wales health patients. January 2012 to March 2024 (seizure data) July to December 2020 (NSW health data).	*N* = 809 falsified alprazolam tablet seizures and 91 notifications of suspected falsified benzodiazepines from New South Wales Health cases	Adinazolam, Bromazolam, Bromonordiazep‐am, Clonazolam, Deschloroetizolam, Diclazepam, Etizolam, Flualprazolam, Flubromazolam, Flubromazepam, Phenazepam, Phenazolam	Falsified presentations most identified were ‘Xanax’ and ‘Mylan’, with 406 and 322 samples detected, respectively. In early falsified samples of ‘Mylan’ and ‘Kalma’, etizolam was the predominant substance, but a wider variety of drugs emerged over time. Among all falsified alprazolam tablets, the most detected substances were the NBZs etizolam (*n* = 228, 28%), clonazolam (*n* = 224, 28%), and bromazolam (*n* = 178, 22%), while only 70 samples (8.7%) contained alprazolam.
Blakey, 2021 Queensland [23]	What's in fake ‘Xanax’? A dosage survey of designer benzodiazepines in counterfeit pharmaceutical tablets	Queensland Health Forensic and Scientific Services Apr 2020—Sept 2020	A rapid dosage survey of 46 counterfeit benzodiazepine tablets from 20 police seizures	Etizolam Flubromazolam Flualprazolam	Counterfeit benzodiazepines presented as loose tablets and in bottles resembling genuine pharmaceutical packaging. Etizolam was the most identified compound, greatest dose variation was also observed for etizolam with a range of 0.7–8.3 mg per tablet. Variability in drug content and dosage observed despite visually similar counterfeit tablets.
Wastewater analysis (*n* = 1)					
Bade, 2023 New South Wales and Queensland [45]	Quantification of new psychoactive substances in Australian wastewater utilising direct injection liquid chromatography coupled to tandem mass spectrometry	Discrete Study December 2021 and January 2022	Influent wastewater samples (250 mL) were collected from seven sites in Queensland (*n* = 4) and New South Wales (*n* = 3)	Clonazolam Etizolam	Detections of etizolam and clonazolam, echo findings of recent Australian and international wastewater and toxicology case reports, indicating these NBZs are of significant concern.
Cross‐sectional survey (*n* = 1)					
Grigg, 2023 National [11]	Real or fake? Sourcing and marketing of non‐prescribed benzodiazepines among two samples of people who regularly use illicit drugs in Australia	Drug Trends, IDRS and EDRS 2021	*N* = 485 people reporting recent non‐prescribed benzodiazepine use	*NA*	Most participants obtained substances sold as registered benzodiazepines, mainly diazepam or alprazolam, often via diverted prescriptions, though some may have unknowingly accessed counterfeits. Those using non‐diverted sources were twice as likely to report obtaining alprazolam, while intentional sourcing of novel compounds was rare.

Abbreviations: CNS, central nervous system; ED, emergency department; EDRS, Ecstasy and Related Drugs Reporting System; IDRS, Illicit Drug Reporting System; NBZ, novel benzodiazepines; NCIS, National Coronial Information System; NPS, new psychoactive substances.

^a^ NBZs are listed alphabetically, not in order of most detected. This table does not list NBZ‐associated metabolites as reported by some authors.

Three studies included in Table 1 indicated that most NBZ‐related deaths and emergency department presentations occur among young adult males [3, 42, 43]. Two of the coronial studies included in this analysis reported that other CNS depressants were co‐detected in 100% of NBZ‐related deaths [19, 42], while the coronial study by Darke et al. found such co‐detections in 95% of cases [43].

Of the 14 studies included in Table 1, eight described data collected before and up to the end of 2022, while four reported on data collected after 1 January 2023. Broadly, data reported in Table 1 suggest that through to 2021, etizolam was the most detected NBZ in Australia [4, 19, 23, 39, 42, 43, 45] with studies noting the more recent emergence of bromazolam, clobromazolam, clonazolam, flualprazolam and flubromazolam [3, 4, 19, 23, 24, 38, 39, 41, 45].

Public drug warnings or drug alerts are issued to inform the community about emerging drug‐related threats, promote harm reduction, support professional awareness and coordination, and reduce drug‐related harms [47]. In Australia, the issuance of such warnings is subject to protocols surrounding data analysis, confirmation and multidisciplinary stakeholder liaison, making them a meaningful indicator of emerging public health concerns [47]. Between January 2020 and June 2025, a total of 23 public drug warnings were issued nationwide relating to NBZs. Of these drug warnings, 18 related to detections of NBZs such as clonazolam or bromazolam and 5 related to detections of opioids in products expected to contain NBZs. A total of 18 alerts were issued in relation to products sold as or believed to contain alprazolam. In 11 of these cases, alerts were related to products branded as Xanax, which were found to contain NBZs, reflecting the broader evidence of the circulation of counterfeit Xanax in Australia [23, 41, 46].

The number of warnings issued in relation to NBZs in Australia has increased over the last 5 years. Of the 23 alerts included in Table 2, 11 were issued between January and June of 2025. Most alerts (*n* = 19) were issued in either the Australian Capital Territory or New South Wales. The concentration of alerts across these jurisdictions likely reflects a detection bias stemming from differences in the analytical and administrative capacities of organisations within these jurisdictions to identify and report emerging drug‐related concerns. For example, Australia's first fixed‐site drug checking service was established in the Australian Capital Territory in 2022 [37], and NSW Health operates a sophisticated close to real‐time drug monitoring program with interdisciplinary, interagency and intersectoral collaboration [32]. As indicated in Table 2, nine NBZ‐related public drug alerts were issued by government public health departments, one was issued by a community‐based and led organisation for people who use drugs, and 13 were issued by drug checking services.

**TABLE 2:** *N* = 23 NBZ related public drug warnings issued in Australia between January 2020 to June 2025.

Year and jurisdiction		Agency type	Title	Expected substance/s	Detected or identified substances^a^, ^b^	Reason/s for alert
2025 (*n* = 11)	ACT	DCS + Gov^c^	Protonitazene, bromazolam, alprazolam and diazepam found in crushed partial ‘Xanax’ pill and dangerous drugs found in counterfeit ‘Xanax’ in Canberra	Alprazolam	Protonitazene, **bromazolam**, alprazolam and diazepam found in crushed partial	Substance detection
	ACT	DCS	Ethylbromazolam found in expected alprazolam pills	Alprazolam	**Ethylbromazolam**	Substance detection
	ACT	DCS	Pregabalin and bromonordiazepam found in ‘alprazolam’ pill	Alprazolam	Pregabalin and **bromonordiazepam**	Substance detection
	ACT	DCS	Diazepam, clonazepam, tapentadol, donepezil and denatonium found in ‘diazepam’ pill	Diazepam	**Clonazepam**, tapentadol, donepezil, Denatonium and diazepam.	Substance detection
	ACT	DCS	Bromazolam and clobromazolam found in ‘Xanax’ pill	Alprazolam	**Bromazolam, clobromazolam**, alprazolam	Substance detection
	ACT	DCS	Clonazepam and diazepam found in ‘alprazolam’ pill	Alprazolam	**Clonazepam** and diazepam	Substance detection
	ACT	DCS	Ethylbromazolam and bromonordiazepam found in ‘alprazolam’ pill	Alprazolam	**Ethylbromazolam** **bromonordiazepam**	Substance detection
	QLD	DCS	Novel benzodiazepine found in counterfeit ‘Xanax’ pressed pill and off‐white powder	Alprazolam	**Ethylbromazolam**	Substance detection
	ACT	DCS	Clonazolam found in counterfeit ‘Xanax’ pill	Alprazolam	**Clonazolam**	Substance detection
	ACT	DCS	Bromonordiazepam found in counterfeit ‘Xanax’ pill	Alprazolam	**Bromnordiazepam**	Substance detection
	ACT	DCS	Bromazolam and clobromazolam found in counterfeit ‘Xanax’ sample	Alprazolam	Alprazolam, **bromazolam, clobromazolam**	Substance detection
2024 (*n* = 5)	NSW	Gov	White powder thought to be cocaine found to contain bromazolam and 2C‐B	Cocaine	**Bromazolam**, 2C‐B	Substance detection, one death and two hospitalisations
	ACT	DCS	Synthetic cannabinoid AB‐MDMSBA found in ‘benzodiazepine’ samples	‘**Novel benzodiaz‐epine**’	AB‐MDMSBA	Substance detection
	ACT	DCS	Bromazolam found in two different counterfeit diazepam samples	Diazepam	**Bromazolam**	Substance detection
	NSW	Gov	Fake diazepam tablets found in NSW	Diazepam	**Bromazolam**, paracetamol, caffeine	Substance detection
	SA	Gov	Fake Xanax pills and other illicit drugs in South Australia have been found to contain dangerous novel synthetic opioids and benzos	Alprazolam	Synthetic opioids (unspecified), **novel benzodiazepines** (unspecified)	Substance detection
2023 (*n* = 2)	ACT	DCS	Bromonordiazepam found in ‘Xanax’ sample	Alprazolam	**Bromonordiazepam**	Substance detection
	QLD	Gov	Fake Xanax tablets containing a strong opioid	Alprazolam	Protonitazene	Substance detection and two deaths
2022 (*n* = 3)	NSW	Gov	Fake Kalma alprazolam tablets found to contain strong opioids	Alprazolam	Etodesnitazene and O‐desmethyltramadol	Substance detection
	NSW	Gov	Update on harmful drugs in fake alprazolam	Alprazolam	**Unregistered benzodiazepines** (unspecified)	Substance detection
	VIC	Gov	High potency benzodiazepine tablets	Alprazolam	**Bromazolam, clonazolam, etizolam, flualprazolam, flubromazepam**	Substance detection
2021 (*n* = 1)	NSW	Peer	Fake Xanax tested positive for Fentanyl in Sydney	Alprazolam	Fentanyl	Substance detection
2020 (*n* = 1)	NSW	Gov	Counterfeit alprazolam	Alprazolam	**Etizolam and other benzodiazepines**	Substance detection

Abbreviations: ACT, Australian Capital Territory; DCS, drug checking service, Gov, Government; NSW, New South Wales; Peer, community‐based peer‐led organisations; QLD, Queensland; SA, South Australia; VIC, Victoria.

^a^ Novel benzodiazepines are bolded.

^b^ Terminology from original alerts used throughout.

^c^ Two communications issued by two separate agencies (ACT Health and CanTEST), counted as one alert as each related to the same detection.

As outlined in Table 2, the number of NBZ related public drug alerts increased from less than three alerts per year between 2020 and 2023 to five alerts in 2024 and 11 alerts between January and June of 2025. This increase in NBZ related alerts, corresponded with an increased capacity to issue alerts in Australia; however, it is noteworthy that alerts relating to NBZs comprised 39% (11/28) of all Australian drug alerts issued to the end of June 2025 [48, 49].

### 3.2 Research Aim 2: The Pharmacology of Commonly Detected NBZs in Australia

Knowledge of NBZ pharmacodynamic and pharmacokinetic profiles is still developing, with much of the current understanding based on reports from people who use NBZs [21, 50, 51]. Available data on NBZs commonly detected in Australia, such as bromazolam, etizolam, clonazolam, flualprazolam and flubromazolam, indicate that these drugs act on the GABA_A_ receptor, and share a common mechanism of action, to produce effects consistent with pharmaceutically regulated benzodiazepines [5]. However, the strong sedative amnestic properties of certain NBZs such as clonazolam and bromazolam, even at low doses, have been noted [5, 25, 52]. Little is known about the pharmacological properties of bromazolam; however, it is known to induce amnesia and has been implicated in a spate of overdose deaths in England and Scotland [51].

As outlined in Table 3, the onset of NBZ effects typically ranges between 15 and 60 min [21]. Certain NBZs (clonazolam, etizolam) are shorter acting [21, 56, 59] however, in an Australian clinical case study, clonazolam was also associated with prolonged sedation [41]. Flualprazolam and flubromazolam are long‐acting NBZs [5, 58, 61], with the latter exhibiting two temporally distinct peaks of serum concentration and an elimination half‐life of over 24 h [5, 21].

**TABLE 3:** Oral dose range, onset, time to peak, half‐life and key effects of diazepam, alprazolam and commonly detected NBZs in Australia.

	Customary dose range* (mg)	Onset** (min)	Time to peak (h)	Half‐life (h)	Key effects as reported in cited literature
Diazepam	2–10^a^	15–60* ^a^	*1–2.5* ^a^	*48* ^a^	Anxiolytic effect, sedative, muscle relaxant and anticonvulsant^a^
Alprazolam	0.25–3^b^	15–30* ^b^	*1–2* ^b^	*10–12* ^b^	Potent anxiolytic, euphoric and disinhibitory effects^b^
Bromazolam	0.5–4^c^	15–45^c^	*Unknown*	*Unknown*	Limited formal data however experiential reports of disorientation and amnesia ^c,l^
Clonazolam	0.2–1^d^	10–30^e^	*Unknown*	3.6^d^	Very potent; and accentuated respiratory depression, dizziness, and muscle relaxation. Experiential reports highlight amnesic effects^e,j^
Etizolam	0.25–3^d^	10–40^l^	0.5–2^f^	3.4–7.1^d^	High potency as an anxiolytic. In small doses, etizolam does not appear to be more harmful than other benzodiazepines currently prescribed in Australia^i^ euphoric effects widely reported^j^
Flualprazolam	0.125–2.5^d^	10–30^h^	*Unknown*	9.5–12^d^	Compared to alprazolam, fluorination increases volume of distribution and overall increased half‐life, volume of distribution and body exposure^k^
Flubromazolam	0.15–0.25^d^	90^g^	3‐10^g^	30^d^	Sedation, amnesia, long‐acting, multiple peaks in serum concentration, hypnotic and amnesic effects reported^j^

*Note:* **a** B Dhaliwal JS, 2023 [53]; **b** George T, 2023 [54]; **c** Crew 2000, 2023 [26]; **d** Yu X, 2023 [21]; **e** Orsolini, 2020 [55]; **f** Drug Enforcement Division, 2025 [56]; **g** Huppertz, 2017 [57]; **h** Drug Enfocement Division, 2025 [58]; **i** Nielsen, 2020 [59]; **j** El Balkhi, 2020 [52]; **k** Canfield, 2023 [60]; **l** Francis, 2024 [25].

* Customary defined here as a dose that is typically used.

** Onset time is reported for oral rather than intravenous doses.

Table 3 provides an overview of the available pharmacokinetic data and effects of the NBZs most frequently detected in the Australian context. Noting that diazepam and alprazolam are not NBZs, we include them in the first two rows of Table 3 as a comparative reference point. Although clobromazolam is increasingly being detected in Australia [19, 38, 42], it is not included in Table 3 due to the absence of published pharmacological or experiential data at the time of writing [21, 38]. Table 3 includes data from in vitro, in vivo and clinical case studies and user reports.

### 3.3 Research Aim 3: Managing NBZ Toxicity, Dependence and Withdrawal

Three studies included in Table 1 were clinical case series, detailing responses to acute NBZ‐related toxicity. The defining clinical characteristics of patients presenting to Australian emergency departments after confirmed NBZ exposure were a prolonged period of sedation—sometimes beyond 24 h [24, 41]. Sedation can progress to more severe outcomes such as coma and respiratory depression. Death after exposure to benzodiazepines without the presence of other drugs is rare [62], while the danger is significantly heightened when benzodiazepines are taken with other CNS depressants due to synergistic effects that can exacerbate sedation and respiratory compromise [43, 63]. In such cases, monitoring and management of airways, respiration and blood flow are generally appropriate as is ruling out alternative or concurrent diagnoses [64]. In more severe presentations, assisted ventilation may be required to sustain respiratory function [63].

The benzodiazepine antagonist flumazenil can reverse the effects of benzodiazepine overdose; however it is not recommended for routine use and should only be considered in severe cases of lone benzodiazepine toxicity, without known contraindications [64, 65]. A meta‐analysis of 13 randomised controlled trials involving 990 patients in which flumazenil administration was compared to placebo, showed that adverse events such as agitation and gastrointestinal symptoms occurred in 27.7% of flumazenil‐treated patients compared to 9.6% with placebo. Serious adverse events such as convulsions and supraventricular arrhythmia were nearly four times more common with flumazenil [66]. Unlike the opioid antagonist naloxone, which is suitable for community administration [67], the risk of adverse events associated with flumazenil warrants medical supervision; as such its roll out to community settings is presently unlikely.

NBZs are believed to pose a higher risk of dependence than prescription benzodiazepines because they often produce stronger effects and can more rapidly lead to tolerance [5, 21, 68]. With regard to pharmaceutically manufactured prescribed benzodiazepines, Australian clinical guidelines provide direction on the assessment and management of dependence and withdrawal [7]. These guidelines, along with the broader evidence base, recommend the use of DSM‐5 criteria, the *Severity of Dependence Scale* or the *Clinical Institute Withdrawal Assessment‐Benzodiazepines* assessment tool to assess benzodiazepine‐related concerns [25, 69, 70]. Treatment options include prescribing interventions, pharmacotherapies, dose tapering, psychotherapies and residential treatment services [70]. However, little is known about the extent to which guidelines and evidence surrounding benzodiazepine treatments are applicable or effective in the context of NBZs.

Managing dependence and withdrawal from NBZs can present unique challenges. One key issue is that the poorly defined pharmacodynamic and pharmacokinetic profiles of NBZs make it difficult to predict the onset, intensity and duration of withdrawal symptoms [25, 71]. This uncertainty complicates clinical decision‐making, particularly when determining whether someone has been using a short‐acting NBZ, which may prompt rapid withdrawal symptoms, or a long‐acting variant, which could delay symptom onset [25]. Moreover, because reliable potency conversions between NBZs and prescribed benzodiazepines are unavailable, determining an appropriate tapering schedule is difficult and, as a result, those who use NBZs could require inpatient/residential withdrawal management [71].

Challenges detecting NBZs further complicates care. Many NBZs do not cross‐react with standard urine drug screening immunoassays. While more sophisticated tests like liquid chromatography–mass spectrometry can identify specific NBZs, they are expensive, slow and not widely available [71]. As such, people seeking treatment for use of NBZs alongside other drugs, may have their NBZ use overlooked.

Compounding these barriers is a lack of clinical service confidence; internationally, reports from people who use NBZs have indicated that some treatment providers might refuse to provide care due to unfamiliarity with appropriate tapering strategies [68]. In such cases, individuals may seek out information online about how to self‐manage a withdrawal [68]. Collectively, the scant evidence base on the management of NBZ dependence and withdrawal highlights the pressing need for an evidence base to inform approaches to care for people who report using illicitly manufactured NBZs.

### 3.4 Research Aim 4: Identifying Harm Reduction Approaches to NBZs

Drawing on the broader findings of this review, along with evidence from NBZ‐related drug alerts, harm reduction resources, and clinical practice guidelines for pharmaceutical benzodiazepine use [7, 25, 72, 73], we have developed a table of key harm reduction education messages. Table 4 uses plain language and is intended as a standalone resource that clinicians and service providers may refer to. It aims to raise awareness about NBZs and provide clear, actionable harm reduction strategies that may reduce the risks associated with the use of benzodiazepines sourced from unregulated markets. These strategies relate to preventing overdose, understanding tolerance and dependence, and managing withdrawal.

**TABLE 4:** NBZs and tailored harm reduction education, talking points for clinicians and other service providers.

Novel benzodiazepine and counterfeit awareness	Overdose prevention
Novel benzodiazepines (NBZ) are drugs that are like prescription medications such as Valium but are not approved for medical use. They are often made and sold illegally, and it is hard to know how strong they are or what's really in them.	**Start low and go slow** on every occasion of use especially with unfamiliar or potentially counterfeit substances.
NBZs can be stronger than prescribed benzodiazepines and even within the same batch, their dosage and potency can vary. This means that even if you test an NBZ at a drug checking service, you may not be able to get precise information about an entire batch.	**Avoid mixing:** Combining benzodiazepines and/or NBZs with other central nervous system depressants (for example, GHB, alcohol, heroin or other opioids) increases overdose risk.
If a benzodiazepine was sourced from anywhere other than a pharmacist, it is likely a fake/counterfeit and could contain NBZs.	**Track doses:** Benzodiazepines or NBZs can reduce inhibitions and cause amnesia, meaning that it is easy to forget if you've already taken a dose.
**Fake, counterfeit and NBZs:**	
Are sometimes made to look like real medications, such as Xanax, Kalma, or Mylan and can be convincing in their press and packaging. Sometimes called “street Xanax” or “street alprazolam”, these may contain NBZs.	**Consider delayed and long‐lasting effects:** It can take up to two hours to feel the effects of certain benzodiazepines or NBZs, some last for over 24 h. Be mindful of delayed effects and prolonged effects before redosing or taking any other substances.
Can contain unknown drugs and unsafe amounts, even if they don't include NBZs.	**Carry and use naloxone:** Benzodiazepines sourced from anywhere other than a pharmacy may contain NBZs and/or opiates. Naloxone won't reverse the effects of benzodiazepines or NBZs but will reverse the effects of opiates and will not cause additional harm.
	**Avoid using alone:** Where possible, use with trusted individuals and stagger your use so that if someone becomes unwell, others are available to call for help.

Tolerance and dependence	Withdrawal
The more often you use, the more your body gets used to it, which means you might need more to feel the same effect.	Depending on whether you are taking short or long‐acting benzodiazepines, withdrawal symptoms may appear within days to a week of stopping benzodiazepine or NBZ use.
**Track your use:** Writing down when and how much you take can help avoid accidental double dosing and give your insight into your tolerance patterns.	**Don't stop suddenly:** If you've been using benzodiazepines or NBZs regularly withdrawal can be dangerous and even life‐threatening.
**Take breaks:** If you're using benzodiazepines or NBZs regularly, spacing out and having days off can reduce risk of dependence.	**Seek support:** A GP or other services may be able to help manage withdrawal symptoms like anxiety, insomnia, shaking, nausea, and even seizures. These can appear quickly if you stop suddenly.
**Know the signs of dependence:** Needing more to get the same effect, feeling anxious without it, or using just to feel normal can be warning signs.	**Taper slowly:** Reducing your dose over time is the safest way to come off benzodiazepines or NBZs. Seek professional guidance on how to safely taper dosing.

## 4 Discussion

This review outlines the detection of NBZs in Australia from 2020 onwards, noting disparate rates of NBZ‐associated deaths among young males [42, 43] and the frequent co‐detection of NBZs alongside other CNS depressants in emergency presentations and coronial investigations [39, 43, 50]. While NBZs have been detected in Australian coronial investigations of drug‐related mortality since 2015 [42, 43], recent coronial reviews indicate escalating numbers of NBZ‐associated deaths since 2019 [19, 42]. Coronial evidence accords with the frequent detection of NBZs in Australian emergency department toxicological monitoring programs [3, 27, 50]. Moreover, our findings demonstrate the increasing frequency of NBZ‐related public drug alerts since 2020 [31]. These findings align with international drug monitoring data. In 2024, the United Nations Office on Drugs and Crime reported that benzodiazepine‐type NPS were the most frequently reported substance group across post‐mortem cases, clinical admissions, and drug driving incidents. Among them, bromazolam was the most commonly identified benzodiazepine‐type NPS in post‐mortem toxicology cases and was frequently detected alongside fentanyl [18].

Most studies included in this review analysed NBZ detections within administrative or analytically confirmed datasets. Our findings indicate that no recent Australian qualitative studies have been conducted among people who source benzodiazepines from unregulated markets. As such, there is limited evidence on the motivations, contexts, and experiences of unregulated benzodiazepine use. This review highlights an imperative for qualitative work to explore consumers awareness of counterfeits, risks associated with NBZs, their harm reduction knowledge and practices.

One Australian survey conducted among people reporting recent stimulant use explored motivations for non‐prescribed benzodiazepine use. Findings indicated that 42% used these drugs to sleep or ‘come down’ after other drugs, 40% for fun or relaxation, 35% to alleviate anxiety or panic, while 31% used them for support with insomnia [74]. To some extent, these findings echo findings from American qualitative research among people seeking substance use treatment related to benzodiazepines, in which participants reported using benzodiazepines to cope, manage the effects of other substances, for sleep, for recreation, to prevent withdrawal, and to socialise [75]. Further, a qualitative study from the United Kingdom explored patterns of concurrent benzodiazepine and opioid use, identifying themes related to anxiety and sleep management, as well as those linked to the pursuit of euphoria or escapism [76].

Some studies included in this review suggested that the up‐scheduling of alprazolam, the introduction of benzodiazepine prescribing guidelines, and the introduction of the Real Time Prescription Monitoring Program may lead people to non‐prescribed use [3, 11]. Similar concerns have been flagged in international research. For example, a content analysis of NBZ‐related online forums indicated that those who knowingly use NBZs report using these drugs after being denied a prescription [68]. This study also highlighted the challenges such people face with regard to dependence and their inability to access appropriate withdrawal support [68]. This reflects wider concerns regarding appropriate withdrawal treatment protocols in a context of limited pre‐clinical and clinical data on NBZs [3, 71].

### 4.1 Limitations

Our findings should be interpreted with regard for several limitations. We chose a narrative methodology because of the breadth of our aims and the range of questions we sought to address, traversing recent Australian evidence, pharmacological profiles of NBZs, and issues in clinical management and harm reduction. This approach allowed us to flexibly incorporate diverse sources which we believe will increase the utility of this review as a resource for clinicians, community organisations and other service providers. However, although we systematically searched for relevant literature and extracted data, our methodology is less rigorous than that of a systematic review. Our identification of the most commonly detected NBZ compounds in Australia was based on aggregate summaries from recent monitoring studies rather than formal quantitative analysis. Finally, the pharmacological profiles we present are limited by the evolving nature of these compounds and the scarcity of available data. For example, Table 3 does not include information on clobromazolam, on which data is yet to be consolidated. Given data limitations, dose ranges for novel benzodiazepines should be interpreted cautiously.

## 5 Conclusions

This review highlights the ongoing presence of NBZs in Australia and the associated harms reflected in related emergency department presentations and deaths reported to the Coroner. While NBZs generally produce effects like pharmaceutical benzodiazepines, many are more potent, with unknown or inconsistent dosages, and are poorly understood in terms of their pharmacodynamics and pharmacokinetics. These gaps in knowledge present significant challenges for harm reduction education and withdrawal management. While this narrative review has focused on monitoring data and drug alerts from Australia, NBZs represent an emerging concern globally [18]. As such, our synthesis of evidence on pharmacology and harm reduction approaches is of international relevance. Future research should focus on improving our understanding of NBZ pharmacology, addressing the risks posed by counterfeit substances, and exploring the lived/living experiences, knowledge, and harm reduction strategies of those who use unregulated and illicitly manufactured benzodiazepines.

## Author Contributions

J.F. led conceptualisation, research, data extraction, writing – original draft. H.M. and S.K. supported research, data extraction and writing, reviewing and editing. K.J.S. supported conceptualisation, data extraction, supervision, writing, reviewing and editing. N.E., K.F. and L.A. supported data extraction, writing, reviewing and editing. A.P., R.S., C.F., J.S. and E.F. supported writing, reviewing and editing.

## Funding

This study was supported by National Drug and Alcohol Research Centre and the National Centre for Clinical Research in Emerging Drugs which is funded by the Australian Government Department of Health, Disability and Ageing under the Drug and Alcohol Program. A.P. is supported by a National Health and Medical Research Council Investigator Fellowship (#1174630). No COIs/constraints on publishing. R.S. is supported by a National Health and Medical Research Council Investigator Fellowship (#1197241).

## Conflicts of Interest

The authors declare no conflicts of interest.

## Supporting information


**Table S1:** Search Terms, PubMed Novel Benzodiazepines Narrative Review.

## Data Availability

Data sharing not applicable to this article as no datasets were generated or analysed during the current study.
