# Supplementary material for: The Emergence of Novel Benzodiazepines in Australia, Evidence, Alerts, Clinical Management and Harm Reduction—A Narrative Review
Source: Drug Alcohol Rev. 2025 Dec 7;45(1):e70086. doi: 10.1111/dar.70086 (PMC12682436; doi:10.1111/dar.70086)
Supplement: Supplementary file 1 — Table S1: Search Terms, PubMed Novel Benzodiazepines Narrative Review. [file DAR-45-0-s001.docx]

**Supporting Information**

Table S1: Search Terms, PubMed Novel Benzodiazepines Narrative Review

| **#** | **Query** |
| --- | --- |
| 1 | benzodiazepine*.mp. |
| 2 | Novel benzodiazepine*.mp. |
| 3 | designer benzodiazepine*.mp. |
| 4 | Signal detection.mp. |
| 5 | early warning system*.mp. |
| 6 | surveillance.mp. |
| 7 | drug alert*.mp. |
| 8 | monitoring/ or monitoring.mp. |
| 9 | toxicology.mp. or toxicology/ |
| 10 | emergency medicine.mp. or emergency medicine/ |
| 11 | drug checking.mp. |
| 12 | Australia.mp. |
| 13 | ("South Australia" or "New South Wales" or "Victoria" or "Queensland" or "Northern Territory" or "Western Australia" or "Tasmania" or "Australian Capital Territory").ab,in,ti. |
| 14 | 1 or 2 or 3 |
| 15 | 4 or 5 or 6 or 7 or 8 or 9 or 10 or 11 |
| 16 | 12 or 13 |
| 17 | 14 and 15 and 16 |
| 18 | limit 17 to yr="2020 - 2025" |
